# Comparison of visual performances of enhanced monofocal versus standard monofocal IOLs in a mini-monovision approach

**DOI:** 10.1186/s12886-023-02920-6

**Published:** 2023-04-21

**Authors:** Tim Beltraminelli, Angelica Rizzato, Katia Toniolo, Andrea Galli, Moreno Menghini

**Affiliations:** Clinic of Ophthalmology, Institute of Clinical Neurosciences of Southern Switzerland (INSI), Ente Ospedaliero Cantonale (EOC), Lugano, Switzerland

**Keywords:** Cataract surgery, Tecnis Eyhance, Mini-monovision, IOL

## Abstract

**Purpose:**

To compare visual performance and quality of life in patients who received either monofocal intraocular lenses (IOLs) or an enhanced monofocal IOL in a mini-monovision target approach.

**Background:**

Monofocal lenses are the most common intraocular IOLs employed during cataract surgery because of their relatively low cost and good performance for distance sight. However, these lenses, generally, do not exonerate patients from spectacle use for near or intermediate tasks. On the other hand, enhanced monofocal IOLs (e.g., Tecnis Eyhance®) feature optical properties providing patients with good intermediate visual outcomes. Satisfactory near visual acuity results, regardless of IOL type, may be achieved through mini-monovision. We assessed visual performance outcomes between these IOLs, in a mini-monovision approach.

**Methods:**

Retrospective case series of patients who underwent bilateral cataract surgery at our institution with implantation of Alcon SN60WF, J&J Tecnis DCB00 or J&J Tecnis Eyhance® DIB00 with a pre-operative mini-monovision target. The postoperative spherical equivalent was measured by a Nidek® auto-refractometer. Best-uncorrected binocular visual acuity (BUBVA) at far (3 m), intermediate (66 cm), and near (40 cm) distance and binocular contrast sensitivity (100%, 25%, and 5%, all at 1 m) were measured using Snellen and Pelli-Robson charts, respectively. Visual performance in daily life was evaluated with the Cataract VF-14 quality of life survey.

**Results:**

71 patients (35 in the monofocal IOL and 37 enhanced IOL group) were enrolled. Patients implanted with enhanced IOL exhibited statistically significant better BUBVA results at 66 cm and 40 cm distances compared to patients in the monofocal group. Additionally, patients in the enhanced IOL group presented a better contrast sensitivity in lower contrast conditions (5%) than patients with monofocal IOL. The quality of life survey showed statistically significant higher scores in daily activities without spectacles for patients with enhanced IOL.

**Conclusion:**

Enhanced monofocal IOLs, combined with a mini-monovision approach, provided patients with good visual performance at all tested distances, with superiority of enhanced monofocal IOLs at near and intermediate distances.

## Introduction

Cataract surgery is the most common surgical procedure in ophthalmology and is currently the only therapeutic intervention for lens opacification [1].

The most widely used approach is implantation of monofocal IOL with an emmetropic target because of the relatively low cost of monofocal lenses and satisfying performances for far vision restoration [2, 3]. This, however, leaves patients undergoing standard cataract surgery with a need for additional correction for intermediate and near vision. Consequently, conferring patients with decreased spectacle dependence has currently become one of the major refractive goals of cataract surgery. Moreover, increased patient demands for a good-quality intermediate vision for daily tasks (e.g., office work or computer usage) has expanded intermediate vision restoration efforts even further.

In Mini-monovision approach the surgeon targets a post-surgical IOL implantation inducing a slight anisometropia, the patient’s dominant eye is targeted for emmetropia, while the non-dominant eye is kept to a slight degree of myopia [4–7]. Thus, the dominant eye is set for far vision, while the non-dominant eye for near vision, hence compensating for presbyopia.

Another strategy is to employ so-called enhanced IOLs, such as the Tecnis Eyhance®. Akin standard IOLs, enhanced monofocal lenses feature an aspheric structure that provides reasonable compensation for far sight. However, the Tecnis Eyhance® IOL possesses a high-order aspheric anterior surface with a continuous change in power from the periphery towards the lens center that has demonstrated better intermediate uncorrected visual acuity [8–11], and higher patient’s satisfaction than classic monofocal IOL [3, 12–15].

In this study, we compared the performances in terms of contrast sensitivity and best-uncorrected binocular vision (near, intermediate, and far) of two conventional models of monofocal IOLs: the Alcon SN60WF and the Johnson&Johnson Tecnis DCB00, with the Tecnis Eyhance® DIB00 from Johnson & Johnson in a mini-monovision surgical approach.

Other studies compared enhanced monofocal IOLs, such as the Tecnis Eyhance® versus standard monofocal IOLs [3, 8, 12–14]. However, to the best of our knowledge, none of the current studies available in the literature compared the visual performances of these intraocular lenses in a mini-monovision setting or tested best-uncorrected binocular visual acuity for these specific IOLs. Additionally, we assessed patients’ subjective quality of life in terms of visual performance, autonomy, and post-surgical spectacle dependence by means of the well-known Cataract VF-14 quality of life questionnaire [16].

## Methods

This is a single-center comparative retrospective study of patients who underwent bilateral standard small-incision phacoemulsification cataract surgery with either implantation of Alcon SN60WF, J&J Tecnis DCB00 or J&J Tecnis Eyhance® DIB00 since 2021.

Patients whose pre-operative refraction target was in the mini-monovision range (i.e., target spherical equivalent (SEQ) of the dominant eye [-0.25 to -0.50 dioptres] and target SEQ of the non-dominant eye [-0.50 to -1.25 dioptres]), whose surgery was conducted by an experienced operator (M.M or A.G.), and who had a documented postoperative follow-up of at least three months were consecutively enrolled and allocated to two groups with respect to the implanted IOL: Group 1 Standard IOL (Alcon SN60WF, J&J Tecnis DCB00); Group 2 Enhanced IOL (J&J Tecnis Eyhance® DIB00). The choice to be implanted with Standard IOLs or Enhanced IOL was made during pre-operative visit by the patient based on his or her preferences after discussion with the surgeon.

Exclusion criteria were the presence of any ophthalmological comorbidity capable of reducing visual potential, failure to give consent, removal of given consent, and unwillingness to undergo additional clinical evaluation for the purpose of the present study.

All patients enrolled were asked to return for various study specific assessments that included: binocular uncorrected vision at far, intermediate and near distance under photopic lighting conditions as well as binocular contrast sensitivity at three different levels of contrast conditions.

All visual acuity and contrast sensitivity tests were done binocularly to recreate daily life conditions. Visual acuity values were measured with Snellen charts at 40 cm, 66 cm, and 3 m with constant room illumination. Visual acuity values are expressed as the logarithm of the minimum angle of resolution (logMAR) for statistical purposes. Contrast sensitivity was measured at 100%, 25%, and 5% contrast using a Pelli-Robson chart at 1 m, according to manufacturer instructions. Values are expressed in terms of the total letter read by the patient. Postoperative spherical equivalent was measured by a Nidek® auto-refractometer. All the measurements were performed by a trained orthoptist (K.T.) who was blinded with respect to the patients’ group assignment.

Quality of life was further assessed by the Cataract VF-14 quality of life questionnaire to obtain patients’ visual performance satisfaction in their daily activities without and with spectacle correction. The survey encompasses most of the daily life activities, and patients express their visual difficulties as “none,“ “a little,“ “moderate,“ “great deal,“ and “unable to do.“ A score is then computed based on their subjective answers [13].

Finally, all patients underwent an ophthalmologic evaluation including intraocular pressure and slit-lamp examination in order to exclude patients who developed ophthalmologic diseases potentially reducing visual acuity.

All patients provided written informed consent, and study was conducted in accordance with Good Clinical Practices and was approved by the Ticino Cantonal ethics committee (Protocol TI3849).

### Statistical analysis

The investigators were not blinded when assessing the results or analyzing the data. Statistical analysis was performed using GraphPad Prism software. Data are shown as mean values ± standard error of the mean (s.e.m.).

## Results

### Patient characteristics and refractive outcome

A total of 71 patients, 34 with standard monofocal—among these, 14 had bilateral SN60WF IOL and 20 bilateral Tecnis DCB00 IOL—and 37 with enhanced monofocal IOL were analyzed.

**Fig. 1:**
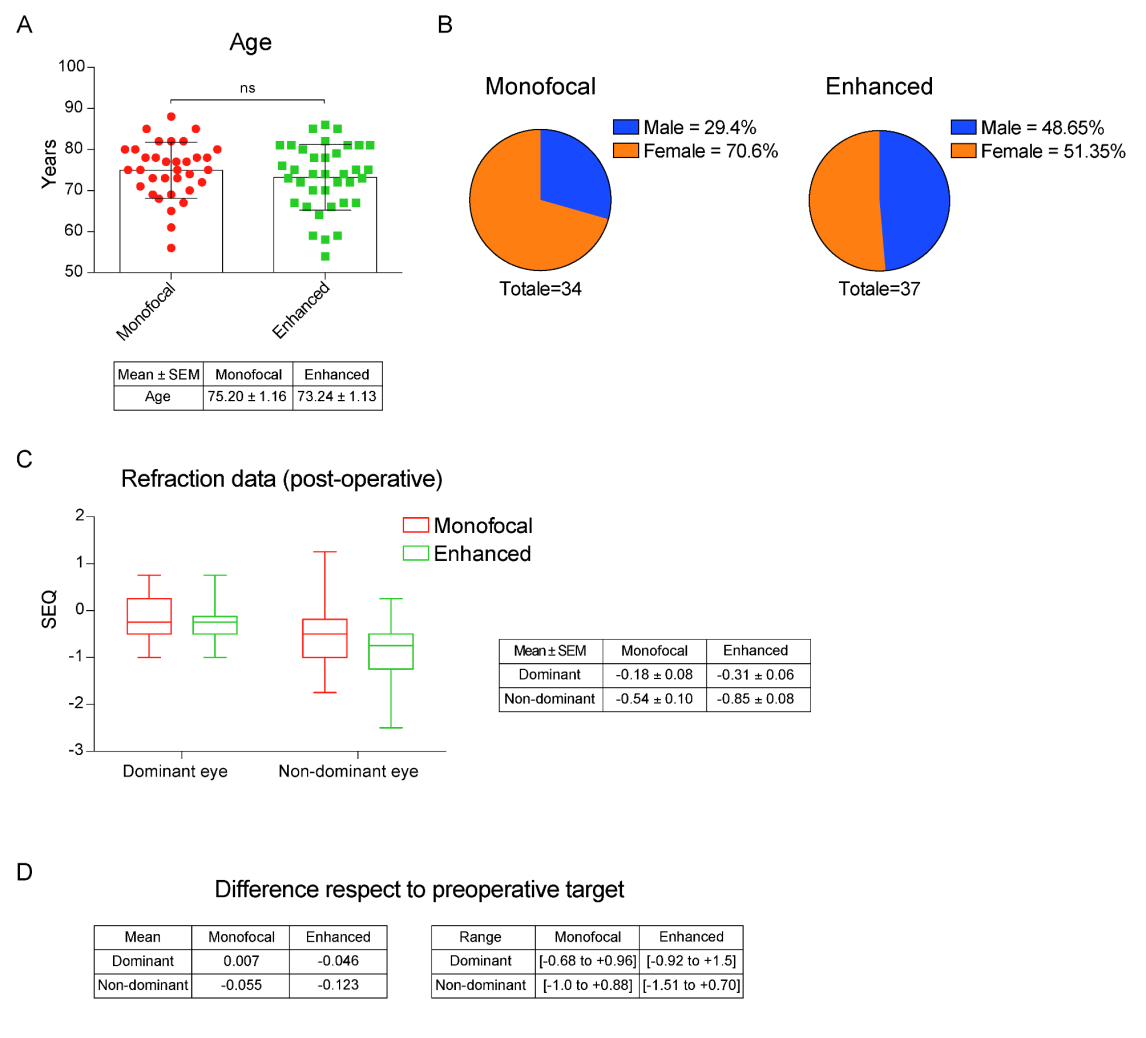
**A)** Histograms of patient age distribution. Data shown as mean ± S.E.M. Test by unpaired t test; p-value = 0.36 **B)** Pie charts of patient gender distribution in both groups **C)** Boxplots of measured post-operative spherical equivalent for dominant and non-dominant eyes in both groups. Table shows data as mean ± S.E.M **D)** Difference in refractive post-operative spherical equivalent compared to pre-operative target for dominant and dominant eye in both groups. Data shows as mean ± S.E.M (left) and range (right)

The two groups were well-balanced in term of age distribution (Fig. 1A-B). Sex distribution exhibited instead a higher percentage of female patients in the standard monofocal (70.6%) compared to the enhanced monofocal group (51.35%).

Postoperative refraction data are shown in Fig. 1C-D. The standard monofocal group showed a mean spherical equivalent (SEQ) of the dominant eye of -0.18 ± 0.08 diopters, of -0.31 ± 0.06 diopters in the non-dominant eye. Mean SEQ values of the enhanced monofocal group were − 0.54 ± 0.10 diopters and − 0.85 ± 0.08 diopters in the dominant and non-dominant eyes, respectively. Statistical analysis showed a statistically significant difference between the two groups in the mean postoperative spherical equivalence of the non-dominant eye (p-value = 0.03, t-test), resulting in more myopic post-operative refraction of non-dominant eyes in the enhanced monofocal group.

### Functional outcomes

**Fig. 2:**
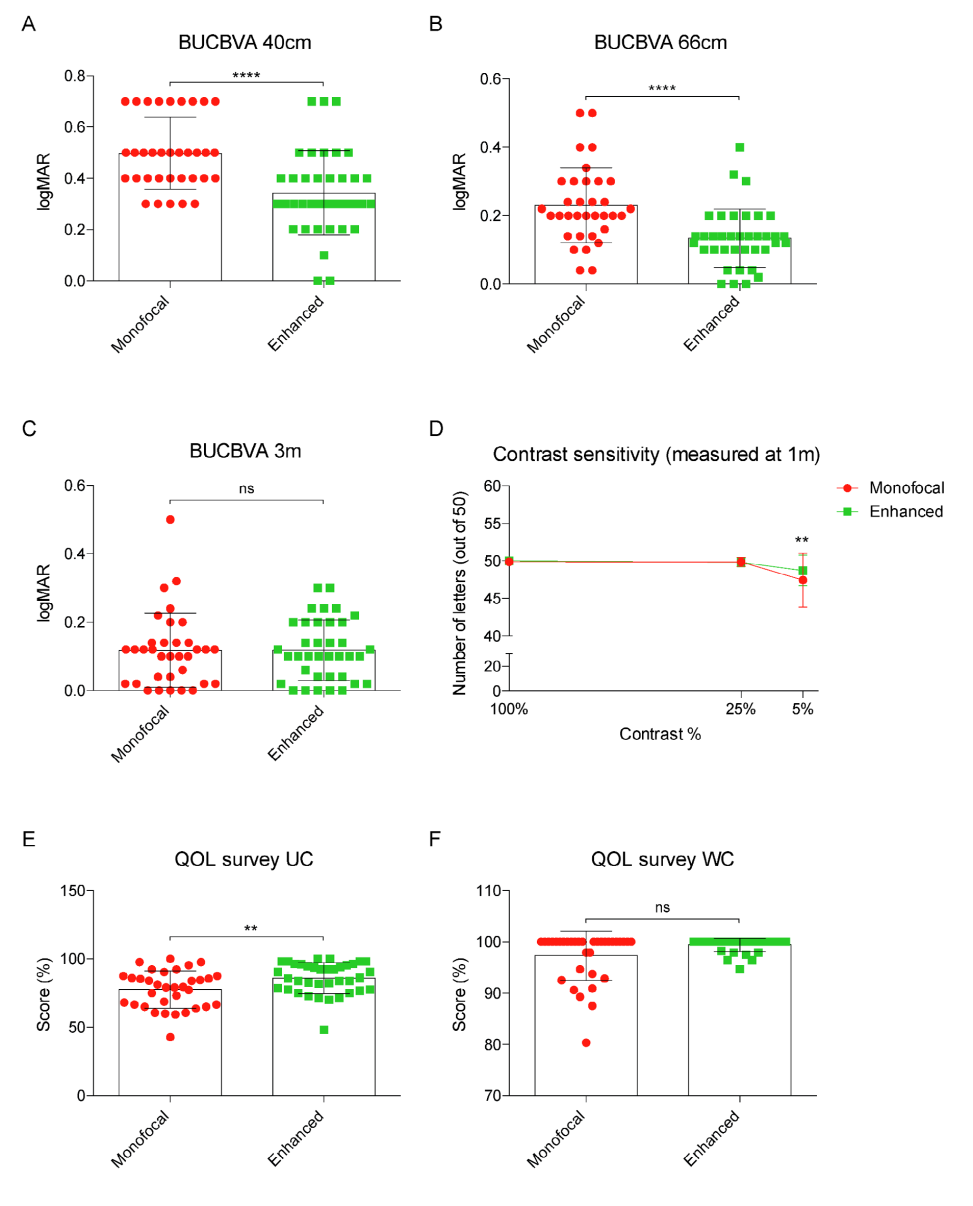
**A)** Histograms of logMAR best uncorrected visual acuities at near (40 cm) distance. Test by Mann-Whitney; p-value < 0.0001 **B)** Histograms of logMAR best uncorrected visual acuities at intermediate (66 cm) distance. Test by Mann-Whitney; p-value < 0.0001 **C)** Histograms of logMAR best uncorrected visual acuities at far (3 m) distance. Test by Mann-Whitney; p-value = 0.81 **D)** Contrast sensitivity measure (1 m) plot. Data shown as number of letter at each contrast percentage tested. Test by 2-way ANOVA with Sidak multi comparison test. P-values (100% = 0.98; 25% = 0.99; 5% = 0.007) **E)** Histograms of VF-14 quality of life survey results in uncorrected tasks. Test by Mann-Whitney; p-value = 0.009 **F)** Histograms of VF-14 quality of life survey results in spectacle-corrected tasks. Test by Mann-Whitney; p-value = 0.09

Visual acuities, tested binocularly and without correction at 40, 66 cm and 3 m are shown in Fig. 2A-C. Mann-Whitney U non-parametric test showed statistically significant better uncorrected binocular visual acuity either at 40 and 66 cm distance (p-value < 0.001 in both cases) for the enhanced monofocal group, whereas no statistically significant difference was observed (p-value 0.81) for uncorrected binocular visual acuity at 3 m between the two study groups. Contrast sensitivity measurement using Pelli-Robson chart was comparable between monofocal and enhanced groups, with the exception of the 5% contrast level, where patients with enhanced monofocal IOL performed slightly better (p-value = 0.007) compared to patients with monofocal IOL (Fig. 2D).

### Quality of life survey

The results of the subjective Cataract VF-14 quality of life patient survey are shown in Fig. 2E-F. Patients implanted with an enhanced monofocal IOL report a statistically significant better subjective quality of life (unpaired t-test, p-value = 0.0068) without the use of spectacles compared to patients who chose the standard monofocal IOL. However, there was no difference when spectacle correction was used for everyday activities, as included in the Cataract VF-14 survey.

## Discussion

We evaluated postoperative spherical equivalent, visual function, and quality of life of a cohort of patients who underwent bilateral cataract surgery with a mini-monovision approach, and implantation of either a standard monofocal intraocular lens (SN60WF or Tecnis DCB00) or so-called enhanced IOL (J&J Tecnis Eyhance®).

Overall, we observed that the refractive values of patients in the enhanced monofocal group were slightly more myopic postoperatively, compared to patients in the standard monofocal group. Similar results were observed in a recent study that showed a wider distribution of post-operative spherical equivalent with a trend towards more myopic outcomes of the J&J Tecnis Eyhance® IOL [3, 13]. The authors suggest that the geometrical anterior surface design of this lens might be responsible [3]. Another theory is that the higher Abbe number provided by Tecnis Eyhance® lenses to compensate for chromatic aberrations, impinges on proper autorefractometer infrared light and accounts for a greater myopic result of objective refraction analysis [17]. The uncorrected binocular distance visual acuity of our enhanced monofocal group indeed implies a refractive power closer to emmetropia rather than myopia. Further studies to better decipher and disentangle the exact optical principles underpinning this phenomenon are warranted.

Our results confirm a superior performance of the enhanced monofocal IOL at intermediate distance (i.e., 66 cm) compared to standard monofocal IOL, and an equal potential at full distance for both IOL models, similar to other recent publications [3, 8, 10, 11, 13].

Analogously to other recent reports, we observed a better uncorrected near vision (40 cm distance) in the group of Eyhance patients [10, 14, 15, 18]. While most likely the mini-monovision setting helps patients in achieving satisfactory results in near vision, it is also believed that Tecnis Eyhance® lenses provide superior reading performance due to a mechanism of neuroadaptation as a consequence of binocular image summation [15], and due to the central defocus in virtue of lens design [10]. The superior intermediate and near vision seen in our cohort could nonetheless be in the context of a slightly more myopic post-operative refraction despite the above mentioned discussion on the typical postoperative refractive artifact of J&J Tecnis Eyhance® IOLs presumably leading to a unbalanced final postoperative refractive status between the two groups. Finally, a selection bias might also have influenced our results as the Tecnis Eyhance® IOLs are presented as premium to the patients, and as such surgeons might be inclined to offer the IOL option to more “*suitable and fitter”* patients.

Our results are corroborated by a recent work by Park et al. in which the visual outcomes and patient satisfaction were compared between patients who bilaterally received the Tecnis Eyhance® IOL either in a mini-monovision setting or with an emmetropic target [11]. Park et al. found similar binocular distance and intermediate visual acuity among the two groups. The mini-monovision setting was however associated with an enhanced reading capability. Unfortunately, due to the absence of a standard monofocal IOL control group, it is not known whether this effect would have been even more marked when compared to standard monofocal IOLs like in our study. Analogously, Gigon et al., reported similar findings comparing patients with Tecnis Eyhance ® to patients with the monofocal Tecnis ZCB00 or a mismatch group [10].

The Tecnis Eyhance®, possesses a physical structure that closely resembles its monofocal alter-ego, with the exception of the high-order aspheric central zone, and as such the Tecnis Eyhance® does not compromise contrast sensitivity to gain depth of focus [9]. Interestingly, our results show even a slightly better performance of the Tecnis Eyhance® IOL at low contrast conditions (i.e. 5%) in comparison to standard monofocal IOLs. We cannot however exclude that this difference is influenced by the non-balanced SEQ between our two cohorts, and a possible influence of the Alcon SN60WF with its blue light filter present in 14 study subjects of our control. Several studies however concluded that there is no difference in contrast sensitivity between an IOL with a blue light filter and such without [19, 20]. Furthermore, Johnson&Johnson Inc. declares better low-light image contrast (30% improvement) compared to Alcon SN60WF as corroborated in the literature [21]. The clinical relevance of the observed difference in contrast sensitivity in our study should however be carefully evaluated. Whereas statistically significant, the magnitude of the difference is fairly small, and the tool used for contrast sensitivity assessment, namely the Pelli-Robson chart, only allows contrast sensitivity to be measured at one single spatial frequency. Despite Pelli-Robson charts being the most widely used test to assess contrast sensitivity [22] and the lack of an globally recognized gold standard, further studies combining different methodologies are warranted to corroborate our results.

Another important aspect of our work is the evaluation of patients’ subjective visual performance satisfaction and spectacle independence through the well-established Cataract VF-14 quality of life questionnaire [16]. In agreement with the literature, we observed that patients in the enhanced monofocal IOL group scored significantly better in this survey when asked how they performed these tasks without spectacle correction than patients in the monofocal IOL group [8]. These results suggest that enhanced monofocal IOLs confer patients with greater spectacle independence and a better subjective visual quality of life compared to monofocal IOLs. In our study, the rates of spectacle independence and patients’ satisfaction in the Eyhance group are likely extolled by the mini-monovision target. Mini-monovision strategy shows high spectacle lens independence in near, intermediate and far vision and higher patients’ satisfaction in previous studies and our findings agree.

Whereas the results of this study are agreement with other recent works, the retrospective design and small sample size of our study is a limitation, and can certainly be a source for bias. Furthermore, a selection bias introduced by the tendency to propose Tecnis Eyhance® IOL to “younger and fitter” patients cannot be argued away as does the impact of an imbalanced refractive status between the two groups on our conclusions.

All these limitations notwithstanding, the strength of this study is represented by the assessment of visual performances by an experimented orthoptists (K.T.) who was blinded with respect to the patient group status. Additionally, we provide real-world, manufacturer-independent data on IOL visual performances. We aimed at recreating as close as possible real life clinical conditions to assess visual function in a spectacle-independent manner.

## Conclusion

In conclusion, our study provides additional real-world data concerning the visual benefit conferred to patients with bilateral implantation of an enhanced monofocal IOLs model, the Tecnis Eyhance®, in a mini-monovision approach. Notably, the superiority of the Tecnis Eyhance® compared to monofocal IOLs manifested particularly at near and intermediate distances, at low-contrast levels, and conferred patients with an enhanced spectacle independence.

## Data Availability

All data analyzed during this study are included in this article. Further enquiries can be directed to the corresponding author.
